# PD123319 Augments Angiotensin II-Induced Abdominal Aortic Aneurysms through an AT2 Receptor-Independent Mechanism

**DOI:** 10.1371/journal.pone.0061849

**Published:** 2013-04-12

**Authors:** Alan Daugherty, Debra L. Rateri, Deborah A. Howatt, Richard Charnigo, Lisa A. Cassis

**Affiliations:** 1 Saha Cardiovascular Research Center, United States of America; 2 Graduate Center for Nutritional Sciences, United States of America; 3 Department of Biostatistics, United States of America; 4 Department of Molecular and Biomedical Pharmacology, University of Kentucky, Lexington, Kentucky, United States of America; Brigham and Women's Hospital, Harvard Medical School, United States of America

## Abstract

**Background:**

AT2 receptors have an unclear function on development of abdominal aortic aneurysms (AAAs), although a pharmacological approach using the AT2 receptor antagonist PD123319 has implicated a role. The purpose of the present study was to determine the role of AT2 receptors in AngII-induced AAAs using a combination of genetic and pharmacological approaches. We also defined effects of AT2 receptors in AngII-induced atherosclerosis and thoracic aortic aneurysms.

**Methods and Results:**

Male AT2 receptor wild type (AT2 +/y) and deficient (AT2 -/y) mice in an LDL receptor −/− background were fed a saturated-fat enriched diet, and infused with either saline or AngII (500 ng/kg/min). AT2 receptor deficiency had no significant effect on systolic blood pressure during AngII-infusion. While AngII infusion induced AAAs, AT2 receptor deficiency did not significantly affect either maximal width of the suprarenal aorta or incidence of AAAs. The AT2 receptor antagonist PD123319 (3 mg/kg/day) and AngII were co-infused into male LDL receptor −/− mice that were either AT2 +/y or −/y. PD123319 had no significant effect on systolic blood pressure in either wild type or AT2 receptor deficient mice. Consistent with our previous findings, PD123319 increased AngII-induced AAAs. However, this effect of PD123319 occurred irrespective of AT2 receptor genotype. Neither AT2 receptor deficiency nor PD123319 had any significant effect on AngII-induced thoracic aortic aneurysms or atherosclerosis.

**Conclusions:**

AT2 receptor deficiency does not affect AngII-induced AAAs, thoracic aortic aneurysms and atherosclerosis. PD123319 augments AngII-induced AAAs through an AT2 receptor-independent mechanism.

## Introduction

Chronic infusion of angiotensin II (AngII) into hypercholesterolemic mice promotes formation of aortic aneurysms that predominantly localize to the suprarenal aortic (AAAs) and ascending aortic (TAAs) regions and augments atherosclerotic lesions [1]–[4]. AngII exerts its bioactive effects through binding to AT1 and AT2 receptors. In rodents, AT1 receptors are expressed as two subtypes termed AT1a and AT1b. AT1a receptors are the most predominant AngII receptor subtype in many tissues of adult mice, whereas AT2 receptors are abundant in fetal development with expression restricted to adrenal glands, brain, kidneys, and heart of adult rodents [5]. The AT2 receptor gene is located on the X chromosome. AngII-induced aortic aneurysms and atherosclerosis are diminished by administration of an AT1 receptor antagonist, losartan, or in mice with AT1a receptor deficiency [4], [6], [7], demonstrating a critical role of AngII-AT1a receptor interaction on aortic pathologies. Despite its low expression in many tissues of adult rodents, AT2 receptors are upregulated during vascular injury [8]. Furthermore, vascular responses to AngII in the absence of AT2 receptors are increased, which are accompanied by upregulation of AT1 receptors [9], [10]. Therefore, it has been proposed that the AngII-AT2 receptor interaction opposes effects of AngII through AT1 receptor activation.

Previous studies have determined roles for AT2 receptors on aortic aneurysms and atherosclerosis using either pharmacological or genetic approaches [6], [11]–[16]. Pharmacological assessments of AT2 receptors in vivo have been limited to the use of a single antagonist, PD123319. While we have reported that PD123319 augments AngII-induced AAAs [6], there are conflicting findings regarding the effects of PD123319 on atherosclerosis. These range from attenuating atherosclerosis in diabetic apolipoprotein (apoE) −/− mice [15], having no effect on AngII-induced atherosclerosis in apoE −/− mice [6], [12], to increased atherosclerosis in young apoE −/− female mice [6] or apoE and AT1a receptor double deficient mice [16]. Since there is always potential for a drug to exert effects through an ancillary effect beyond its class-specific property, it is unclear whether these inconsistencies are due to off-target effects of PD123319. AT2 receptor deficient mice were developed by two independent groups [17], [18]. In agreement with the inconsistent results from PD123319, AT2 receptor deficiency has shown reduction [15], augmentation [13], [14], no effect [11], or no effect on lesion size but influencing cellular and molecular elements in atherosclerotic lesions [14]. No study has determined the role of genetic AT2 receptor deficiency on AngII-induced aortic aneurysms.

In the present study, we defined the role of AT2 receptor deficiency on AngII-induced aortic aneurysms and atherosclerosis. We also infused PD123319 into wild type and AT2 receptor deficient mice to define the specificity of this pharmacological approach on these aortic pathologies.

## Materials and Methods

### Ethics Statement

All mouse studies were performed with approval of the University of Kentucky Institutional Animal Care and Use Committee.

### Mice and Diets

LDL receptor −/− mice of C57BL/6 background were purchased from the Jackson Laboratory (B6.129S7-Ldlr^tm1Her^; Stock # 002207; Bar Harbor, ME). Male AT2 receptor deficient mice were supplied by Dr. Inagami at Vanderbilt University. Since AT2 receptor is located on the X chromosome, male AT2 receptor deficient mice are represented as AT2 -/y, and wild type littermates are denoted as AT2 +/y. Male AT2 -/y mice and female LDL receptor −/− mice were mated, and their offspring were bred to generate LDL receptor −/− mice that were either wild type or deficient for AT2 receptors. Only male mice were used in this study since females are not susceptible to AngII-induced AAAs [19]. All mice were maintained in a barrier facility and fed a normal rodent diet ad libitum.

To induce hypercholesterolemia, mice were fed a diet supplemented with saturated fat (milk fat 21% wt/wt; Diet # TD.88137; Harlan Teklad; Indianapolis, IN). This diet feeding started 1 week prior to pump implantation and was maintained during 4 weeks of infusion. The entire duration of fat-enriched diet feeding was 5 weeks.

### Genotyping and Reverse Transcription Polymerase Chain Reaction (RT-PCR)

AT2 receptor genotypes of mice were confirmed using PCR. AT2 receptor genotyping used the following primers: forward 5′-GTAAGAATTTGGAGTTGCTG, and reverse 5′-GGGATTCCTTCTTTGAGAC. PCR reaction was 35 cycles of 95°C for 1 min, 56°C for 1 min, and 72°C for 2 min; and 1 cycle of 72°C for 5 min. Resultant amplicons for wild type and disrupted alleles were 500 bp and ∼1.1 kb, respectively as shown in Figure S1A. LDL receptor genotyping used the following primers: 5′-AGGTGAGATGACAGGAGATC, 5′-AGGATGACTTCCGATGCCAG, and 5′-GCAGTGCTCCTCATCTGACTTG. PCR reaction was 35 cycles of 95°C for 1 min, 50°C for 1 min, and 72°C for 2 min; and 1 cycle of 72°C for 5 min as described previously.

RNA was isolated from aortic tissues of LDL receptor −/− mice, that were either AT2 +/y or −/y, using the SV Total RNA Isolation System (Catalog # Z3100, Promega Corp, Madison, MI). RT-PCR of AT2 receptor and β-actin was performed using the Access RT-PCR system (Catalog # A1250, Promega Corp) in a thermocycler (Bio-Rad, Hercules, CA, USA) as described previously. Samples containing either no template or no RT reactions were used as negative controls.

### Osmotic Minipump Implantation and AngII Infusion

Osmotic minipumps (Alzet Model 2004; Durect Corp; Cupertino, CA) were implanted subcutaneously to deliver saline, AngII (500 ng/kg/min; catalog # A9525; Sigma-Aldrich; St. Louis, MO), or PD123319 (3 mg/kg/d; catalog # P186, Sigma-Aldrich) as described previously [1], [6].

### Serum Cholesterol Measurement

Serum cholesterol concentrations were determined using commercially available enzymatic assay kits (Catalog # 439-17501; Wako Chemicals; Richmond, VA). Lipoprotein cholesterol distributions were performed by size exclusion chromatography as described previously [20].

### Systolic Blood Pressure Measurement

Systolic blood pressures were measured on conscious, restrained mice using a non-invasive tail cuff system (BP-2000; Visitech; Apex, NC) at the same time of each day, 5 consecutive days every week as described previously [21].

### Aortic Aneurysm and Atherosclerosis Quantification

Aortas were excised at termination and fixed with 10% neutrally buffered formalin. After cleaning off adventitia, maximal aortic widths of suprarenal aortas were quantified by measuring ex vivo maximal diameters of suprarenal aortas using an Image-Pro Plus software (Media Cybernetics; Rockville, MD) as described previously [22]. Definition of AAA incidence included those having ex vivo aortic width at least 50% larger compared to the mean aortic width measured in mice infused with saline as well as death due to aortic rupture located in abdominal aortas.

Atherosclerosis was quantified on intima of the aortic arch region that contained the ascending portion, arch, and part of the descending thoracic portion (from the aortic orifice of left subclavian artery to 3 mm below) as described previously [23]–[25].

### Characterization of Abdominal Aortic Aneurysms

Three suprarenal aortas from each group were chosen based on the mean maximal diameter as shown in Figure S2, embedded in OCT, and serially sectioned using a cryostat. The aneurysm was sectioned (10 µm of thickness) in serial sets consisting of 10 slides with 10 serial sections per slide as described previously [26]. Movat's pentachrome staining was performed using a commercial kit (Catalog # K042; Poly Scientific R & D Corp; Bay Shore, NY) to distinguish elastin fibers and collagen in AAA tissues.

### Statistical Analyses

Version 9.2 of SAS (SAS Institute Inc.; Cary, NC) and SigmaStat (Point Richmond, CA) were used for statistical analyses. Data are represented as mean±standard error of the mean (SEM). Two way ANOVA was performed to compare continuous variables among groups that contained factors including two genotypes (AT2 +/y versus AT2 -/y) and two manipulations (saline versus AngII, or AngII versus AngII+PD123319). Incidence of AAAs was analyzed using Fisher's exact test. Systolic blood pressure data were analyzed using two way repeated measures ANOVA. P<0.05 was considered significant.

## Results

### AT2 Receptor Deficiency Did Not Change Aortic Pathologies in Mice Infused with AngII

Presence or deletion of AT2 receptors in mice was confirmed by AT2 receptor genotyping (Figure S1A) and RT-PCR showing a complete absence of AT2 receptor mRNA in aortic tissues (Figure S1B). Infusion of either saline or AngII (500 ng/kg/min) had no significant effect on systolic blood pressure in either AT2 +/y or AT2 -/y mice (Table 1). A small, but significant, effect of genotype on body weights was detected (AT2 +/y versus AT2 -/y: P = 0.03 in Table 1), but there was no significant effect for manipulation (saline versus AngII infusion) or interaction between AT2 receptor genotype and manipulation. Plasma cholesterol concentrations showed a small but significant difference between AT2 +/y and AT2 -/y mice infused with AngII (P = 0.001; Table 1), whereas there was no significant difference between the two AT2 genotypes infused with saline.

**Table 1:** Characteristics of Mice Infused with either Saline or AngII.

Infusion	AT2 Genotype	N	Body Weight (g)	Plasma Cholesterol (mg/dl)	Systolic Blood Pressure (mmHg)
Saline	+/y	11	29.7±1.1	1438±61	138±5
	−/y	11	32.4±1.2 *	1444±37	136±3
Ang II	+/y	16	29.8±0.4	1330±35	144±4
	−/y	18	30.9±0.8 *	1590±58 ^#^	139±3

Body weights and plasma cholesterol concentrations were determined at termination. Systolic blood pressures were recorded during the last week of the study. Values are represented as mean±SEM. Two way ANOVA was performed for body weights and plasma cholesterol concentrations, and two way repeated measures ANOVA was used to analyze systolic blood pressures. * P = 0.03 between AT2 +/y and −/y genotypes. ^#^ P = 0.001 between AT2 +/y and −/y mice infused with AngII. There were no significant differences between AT2 receptor genotypes (+/y versus −/y) and between manipulations (saline versus AngII) for systolic blood pressures.

**Figure 1:**
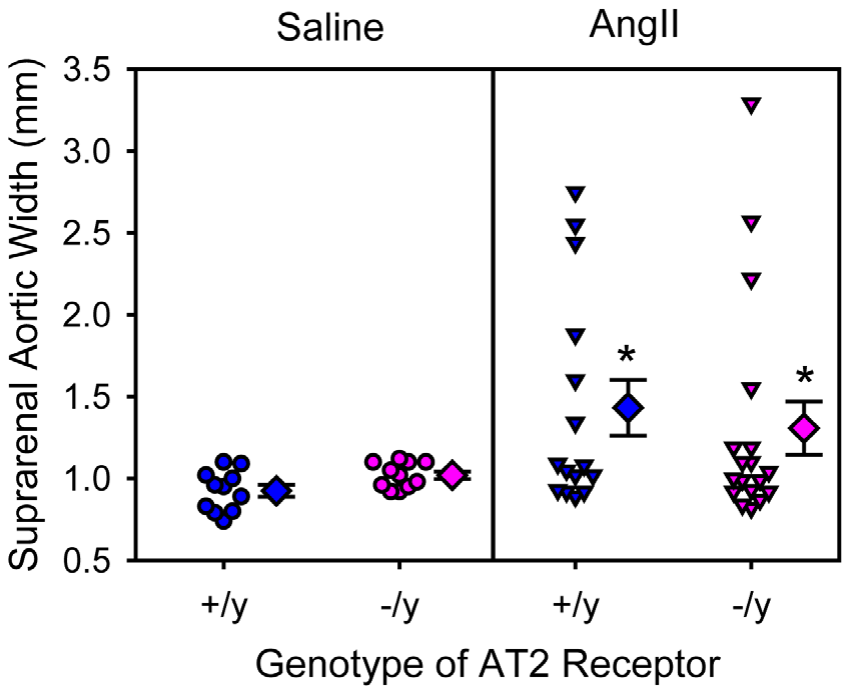
AT2 receptor deficiency did not change suprarenal aortic expansion in mice infused with AngII. Maximal aortic width of suprarenal aortas was measured ex vivo. Circles and inverted triangles represent values from individual mice, diamonds are mean values of each group, and bars are SEM. * P = 0.002 between AngII-infused groups and saline-infused groups as analyzed by two way ANOVA. There were no significant effects of genotype (AT2 +/y versus AT2 −/y) on aortic width.

Consistent with our previous findings [1], [6], infusion of AngII led to significant increases in maximal widths of suprarenal aortas (Figure 1) and incidence of AAAs (saline versus AngII: 0% versus 40%; P = 0.004). AT2 receptor deficiency had no significant effect on AngII-induced AAAs, as determined by both maximal widths of suprarenal aortas (Figure 1) and incidence of AAAs (AT2 +/y versus −/y: 40% versus 28%; P = 0.74). Death due to aortic rupture occurred in 1 of 17 AT2 +/y mice, and in 0 of 18 AT2 −/y mice.

**Figure 2:**
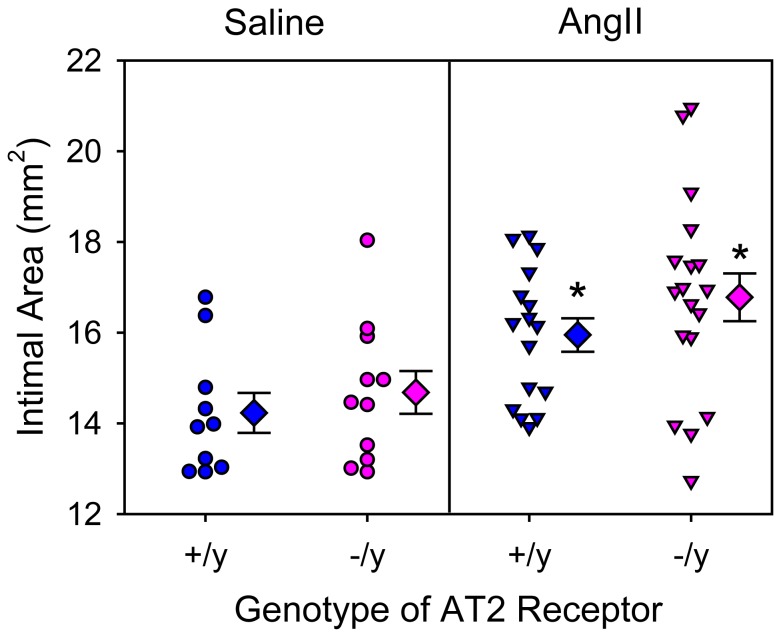
AT2 receptor deficiency did not change expansion of the aortic arch region in mice infused with AngII. Intimal area of aortic arches was calculated from en face measurements. Circles and inverted triangles represent values from individual mice, diamonds are mean values of each group, and bars are SEM. * P = 0.0003 between AngII-infused and saline-infused groups as analyzed by two way ANOVA. There were no significant effects of genotype (AT2 +/y versus AT2 −/y) on intimal areas of aortic arches.

**Figure 3:**
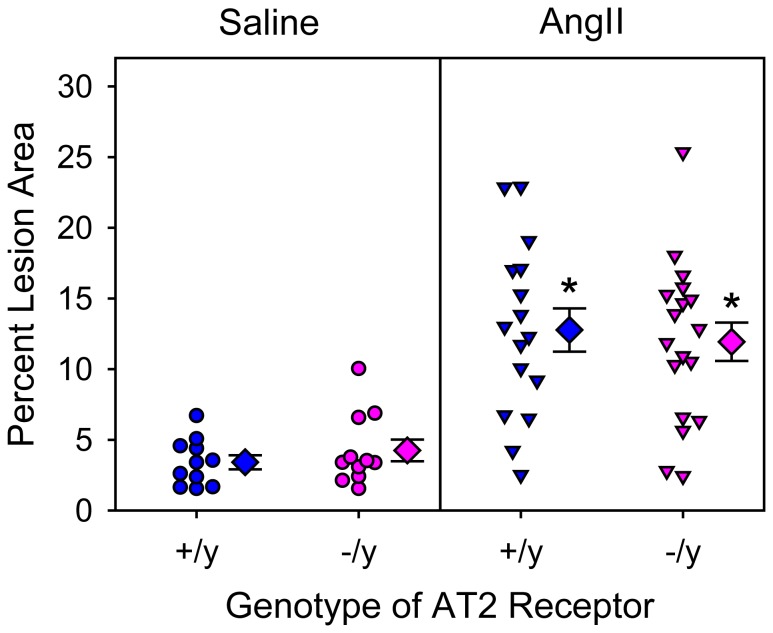
AT2 receptor deficiency did not change percent atherosclerotic lesion area in mice infused with AngII. Percent atherosclerotic lesion area of aortic arches was calculated as lesion area/intimal surface area (x 100%). Circles and inverted triangles represent values from individual mice, diamonds are mean values of each group, and bars are SEM. * P<0.0001 between AngII-infused groups and saline-infused groups as analyzed by two way ANOVA. There were no significant effects of genotype (AT2 +/y versus AT2 −/y) on lesion areas.

In addition to AngII infusion inducing AAAs, we have reported previously that AngII infusion induced TAAs localized to the ascending portion of thoracic aortas and augmented hypercholesterolemia-induced atherosclerosis [1], [3], [4]. Consistent with the previous reports, AngII infusion induced TAAs as defined by increased intimal area of the aortic arch (Figure 2) and augmented atherosclerosis in the same region (Figure 3). However, AT2 receptor deficiency had no significant effect on either of these AngII-induced aortic pathologies.

### PD123319 Augmented AngII-induced AAAs Independent of AT2 Receptor Genotype

**Figure 4:**
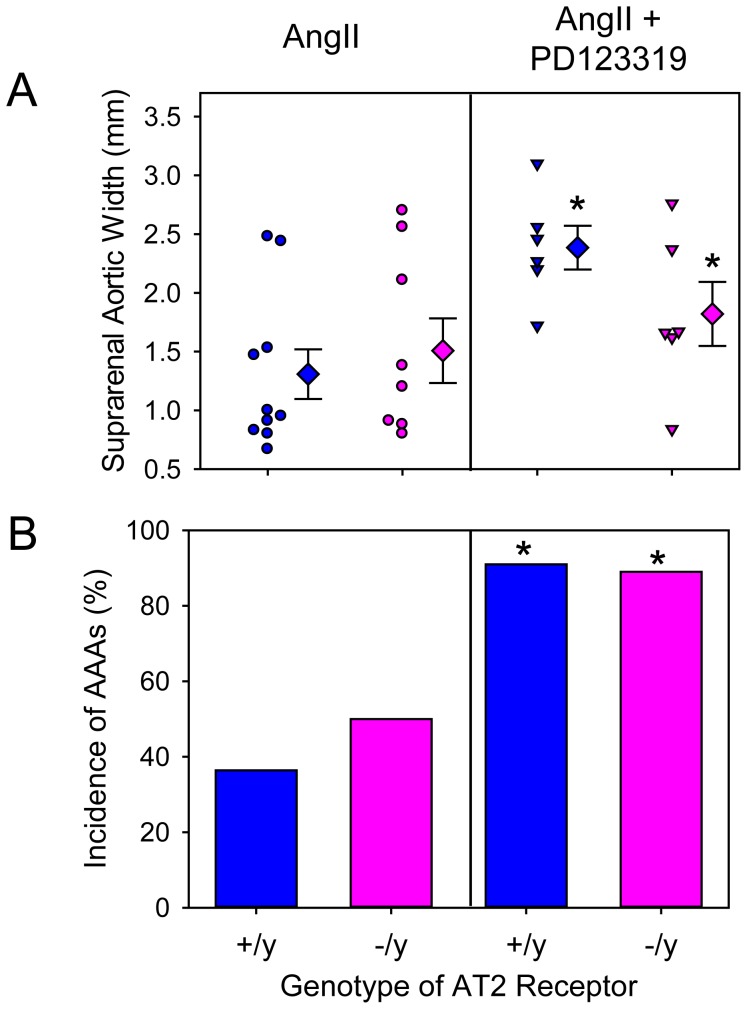
PD123319 augmented AngII-induced suprarenal aortic expansion and incidence of AAAs independent of AT2 receptor genotype. A. Maximal aortic width of suprarenal aortas was measured ex vivo. Circles and inverted triangles represent values from individual mice, diamonds are mean values of each group, and bars are SEM. * P = 0.01 between AngII-infused and PD123319-infused groups as analyzed by two way ANOVA. There were no significant effects on aortic width for genotypes (AT2 +/y versus AT2 −/y). B. Incidence of AAAs. * P = 0.006 between AngII-infused and PD123319-infused groups as analyzed by Fisher's Exact test. There were no significant effects of genotype (AT2 +/y versus AT2 −/y) on incidence of AAAs.

**Figure 5:**
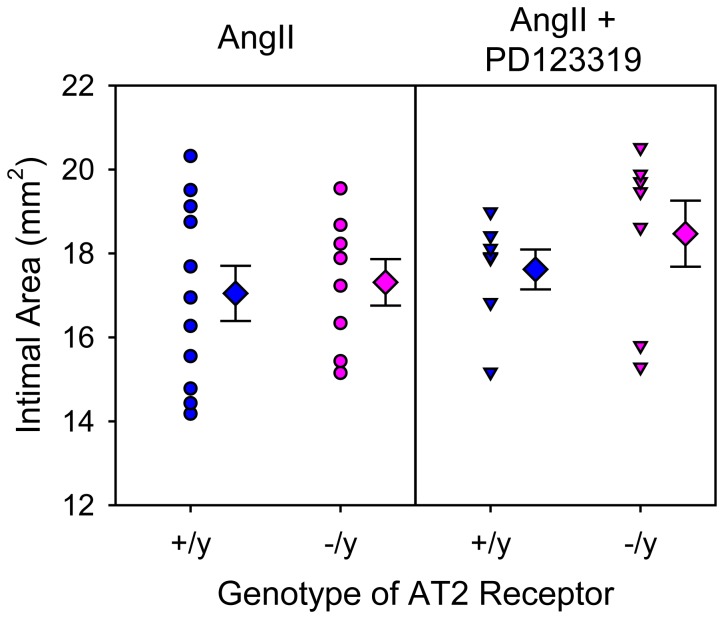
PD123319 did not change AngII-induced expansion of the aortic arch region. Intimal area of aortic arches was measured using an en face method after termination. Circles and inverted triangles represent values from individual mice, diamonds are mean values of each group, and bars are SEM. There were no significant effects of genotype (AT2 +/y versus AT2 -/y) or PD123319 on intimal areas of the aortic arch region, as demonstrated by two way ANOVA.

**Figure 6:**
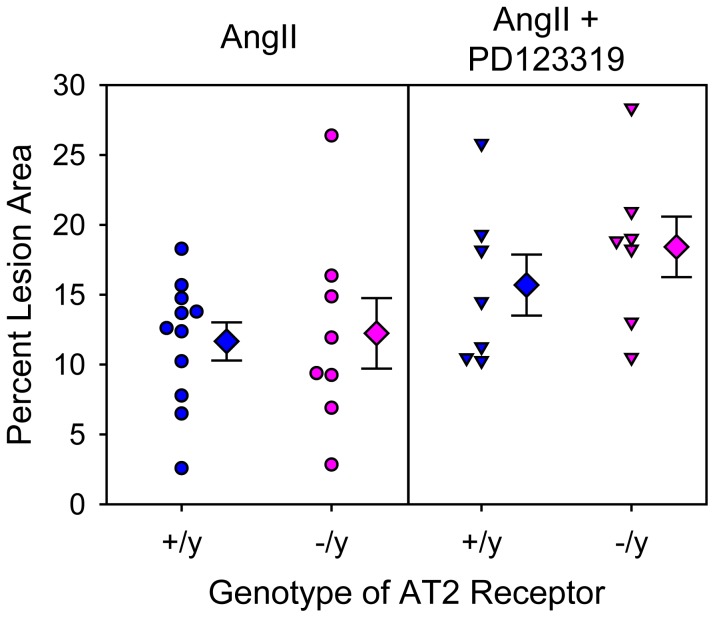
PD123319 did not change atherosclerotic lesion size in mice infused with AngII. Percent atherosclerotic lesion area of aortic arches was calculated as lesion area/intimal surface area (x 100%). Circles and inverted triangles represent values from individual mice, diamonds are mean values of each group, and bars are SEM. There were no significant effects of genotype (AT2 +/y versus AT2 −/y) or PD123319 on lesion areas, as demonstrated by two way ANOVA.

PD123319 is the most commonly used AT2 receptor antagonist to define AT2 receptor effects in vitro and in vivo. As shown in our previous study, PD123319 augmented AngII-induced AAAs [6]. However, while this compound exerts specificity for AT2 receptors in vitro [27], [28], no studies have defined the in vivo specificity of PD123319 by administering the antagonist to AT2 receptor deficient mice. Therefore, we co-infused AngII and PD123319 into male LDL receptor −/− mice that were either AT2 +/y or −/y. Co-infusion of PD123319 with AngII did not significantly change body weights, plasma cholesterol concentrations, or systolic blood pressures in either AT2 +/y or −/y mice (Table 2). However, co-infusion of PD123319 with AngII increased AAAs as demonstrated by both maximal widths of suprarenal aortas and incidence of AAAs, irrespective of AT2 genotype (Figure 4 and Figure S2). There was no significant effect of PD123319 on AngII-induced TAAs (Figure 5) or atherosclerosis (Figure 6) in either AT2 genotype. Rate of death due to aortic rupture in the abdominal region was not significantly different by either AT2 receptor genotype or manipulation (Table 2).

**Table 2:** Characteristics of Mice Infused with AngII alone or both AngII and PD123319.

Infusion	AT2 Genotype	N	Body Weight (g)	Plasma Cholesterol (mg/dl)	Systolic Blood Pressure (mmHg)	Aortic Rupture
AngII	+/y	11	29.8±1.2	1062±44	154±5	0/11
	−/y	11	29.5±1.5	1029±83	160±7	2/9
						
Ang II+PD123319	+/y	8	28.3±0.6	1028±37	152±7	3/10
	−/y	7	26.8±0.9	1151±167	155±6	3/10

Body weights and plasma cholesterol concentrations were determined at termination. Systolic blood pressures were recorded during the last week of the study. Aortic rupture was confirmed by necropsy with visible blood clot in the abdominal cavity and verification of disrupted aortic structure of abdominal aorta. Values are represented as mean±SEM. Two way ANOVA was performed for body weights and plasma cholesterol concentrations, two way repeated measures ANOVA was used to analyze systolic BP data, and Fisher's exact test was done for aortic rupture rate. There were no significant differences between AT2 receptor genotypes (+/y versus −/y) and between manipulations (AngII versus AngII+PD123319) for all parameters tested in this table.

Infusion of AngII at a rate of 500 ng/kg/min promoted limited pathology with minimal disruption of elastin fibers (Figure S3). In contrast, administration of PD123319 greatly enhanced aortic pathology with appearance of transmural elastin breaks of aortic media and aberrant collagen accumulation. Marked heterogeneity of cellular characteristics in AngII-induced AAAs was also evident in mice administered PD123319. We were unable to discern any differences in cellular characteristics of aneurysmal tissues in mice administered PD123319 irrespective of AT2 receptor genotype.

## Discussion

This study, using both genetic disruption and pharmacological inhibition approaches, demonstrated that AT2 receptor deficiency did not influence AngII-induced AAAs, TAAs, and atherosclerosis in hypercholesterolemic mice. Notably, while PD123319 augmented AngII-induced AAAs in AT2 receptor wild type mice, the drug was equally effective in promoting this effect in AT2 receptor deficient mice. This infers that when administered in vivo, PD123319 exerts its effects through a mechanism that is independent of AT2 receptor antagonism.

There is compelling evidence from many laboratories that AngII infusion promotes aortic aneurysms and augments atherosclerosis [1], [3], [4], [29]–[32]. Among the 3 subtypes of AngII receptors in rodents, AT1a receptors promote AngII-induced aortic aneurysms and atherosclerosis as demonstrated by pharmacological inhibition of AT1 receptors [6] and genetic deletion of AT1a receptors [4], [7] in our previous studies. In contrast to the well-defined functional effects of the AngII-AT1a receptor interaction, no study has employed both pharmacological and genetic approaches to defining effects of AT2 receptors on AngII-induced AAAs. Definition of AT2 receptor function in vivo has been limited by the availability of few pharmacological tools. The most commonly used pharmacological reagents are PD123319 as an antagonist and CGP42112 as a partial agonist, respectively. We reported previously that simultaneous administration of PD123319 at the same dose (3 mg/kg/day) used in the present study with AngII infusion into hypercholesterolemic mice exacerbated AAAs [6]. A range of doses (1–50 mg/kg/day) of PD123319 were used to define several different AT2 receptor-dependent effects in mice [28], [33]–[36]. The dose of PD123319 (3 mg/kg/day) used in the present study was similar to the lowest dose used in most mouse studies [6], [12], which minimize the possibility of nonspecific effects of this compound. Despite this low dose, AT2 receptor independent effects of PD123319 on AAAs, but not on atherosclerosis and systolic blood pressure, were demonstrated in AngII-infused mice lacking AT2 receptors. There are also reports that 5 mg/kg/d of PD123319 decreased atherosclerosis in diabetic apoE −/− mice [15], while 30 mg/kg/d of PD123319 did not affect systolic blood pressure in C57BL/6 mice infused with AngII 500 ng/kg/min [37]. These findings indicate that there might be dose-dependent responses for off-target effects of PD123319 on different parameters.

The ablation effect of AT1a receptor deficiency on AngII-induced AAAs and augmentation of this aortic pathology by PD123319 implied that AT2 receptors may antagonize AT1a receptor-mediated pathophysiological roles of AngII. Given previous findings [6], these results prompted us to test whether PD123319 increases AAAs through an AT2 receptor-dependent mechanism. Consistent with our previous study [6], co-infusion of PD123319 with AngII led to augmentation of AAAs. Additionally, as demonstrated by Movat's pentachrome staining, PD123319 administration led to severe destruction of the aortic wall. However, this effect was comparable between the two AT2 genotypes, inferring that PD123319 augmented AngII-induced AAAs through an ancillary mechanism that is independent of AngII-AT2 receptor interaction. To support the findings from the present study, a previous study has provided evidence that PD123319 acts through an AT2 receptor-independent mechanism in AngII-induced vasoconstriction in mice [38]. Although PD123319 significantly increased AngII-induced AAAs independent of AT2 receptors, the complexity of the aneurysmal response will provide a challenge in determining the mechanism of this effect [36], [39], [40].

In addition to AAAs and atherosclerosis, chronic AngII induces TAAs that are restricted to the ascending aortic region [3], [4]. The role of AngII receptors has also been inferred in another mouse model with TAAs that is provoked by mutation of fibrillin-1 [41], [42]. Losartan, an AT1 receptor antagonist, reduced TAAs in this mouse model with Marfan syndrome [41], whereas AT2 receptor deficiency augmented aortic expansion in this mouse model [42]. These findings indicate that AT2 receptors may prevent aortic expansion through antagonizing AngII-AT1 activation. For comparison to these reports, we also defined the role of AT2 receptors on AngII-induced TAAs in the present study. As noted previously, AngII infusion induced TAAs in LDL receptor −/− mice irrespective of AT2 genotype. In contrast to AngII-induced AAAs, neither AT2 receptor deficiency nor PD123319 significantly affected AngII-induced TAAs. Therefore, unlike TAAs associated with fibrillin-1 mutations [42], our results do not support a role of AT2 receptors in AngII-induced TAAs.

In the current study, the role of AT2 receptors on AngII-induced atherosclerosis was determined in male LDL receptor −/− mice. AT2 receptor deficiency and PD123319 administration have been used in several studies in apoE −/− mice to determine effects on atherosclerosis with variable results [12]–[15], [34]. In the current study, neither deficiency of AT2 receptors nor PD123319 administration (separately or concomitantly) had any effect on atherosclerotic lesion size. Therefore, this study provides further confirmation for a limited role of AT2 receptors in atherosclerosis.

In conclusion, AT2 receptor deficiency has no effect on either aortic aneurysms or atherosclerosis. PD123319, despite being accepted as a specific AT2 antagonist, augments AngII-induced AAAs through an AT2 receptor-independent mechanism.

## Supporting Information

Figure S1 **Verification of AT2 receptor deficiency in AT2 -/y mice.** (**A**) Genotypes of the AT2 receptor were determined with PCR. Primers (arrows in cyan color) were located upstream and downstream of the neo sequence in exon 3 of the AT2 receptor gene, respectively. Amplicon sizes of wild type and disrupted alleles were 500 bp and ∼ 1100 bp, respectively. (**B**) mRNA of AT2 receptor (160 bp) and β-actin (236 bp) was determined by RT-PCR. AT2 receptor mRNA was detectable in extracts of aortas from AT2 +/y mice, but not from AT2 -/y mice. No RT was used as a negative control.
(PDF)

Figure S2 **Ex vivo images of abdominal aortas.** After removal of adventitia, aortas were pinned for photography. Ex vivo diameters were measured using ImagePro Plus software. These selected images represent outer diameters of suprarenal aortas near the mean value of each group.
(PDF)

Figure S3 **Pathology of abdominal aortic aneurysms.** Suprarenal aortas were sectioned and stained to determine tissue characteristics. Movat's pentachrome staining was performed to visualize structures of aortic tissues. The images of representative sections demonstrate the similar heterogeneity of aneurysmal tissue pathologies in mice infused with PD123319 irrespective of the AT2 genotype.
(PDF)
