# Supplementary material for: PD123319 Augments Angiotensin II-Induced Abdominal Aortic Aneurysms through an AT2 Receptor-Independent Mechanism
Source: PLoS One. 2013 Apr 12;8(4):e61849. doi: 10.1371/journal.pone.0061849 (PMC3625148; doi:10.1371/journal.pone.0061849)
Supplement: Figure S1 — Verification of AT2 receptor deficiency in AT2 -/y mice. (A) Genotypes of the AT2 receptor were determined with PCR. Primers (arrows in cyan color) were located upstream and downstream of the neo sequence in exon 3 of the AT2 receptor gene, respectively. Amplicon sizes of wild type and disrupted alleles were 500 bp and ∼ 1100 bp, respectively. (B) mRNA of AT2 receptor (160 bp) and β-actin (236 bp) was determined by RT-PCR. AT2 receptor mRNA was detectable in extracts of aortas from AT2 +/y mice, but not from AT2 -/y mice. No RT was used as a negative control. (PDF) [file pone.0061849.s001.pdf]

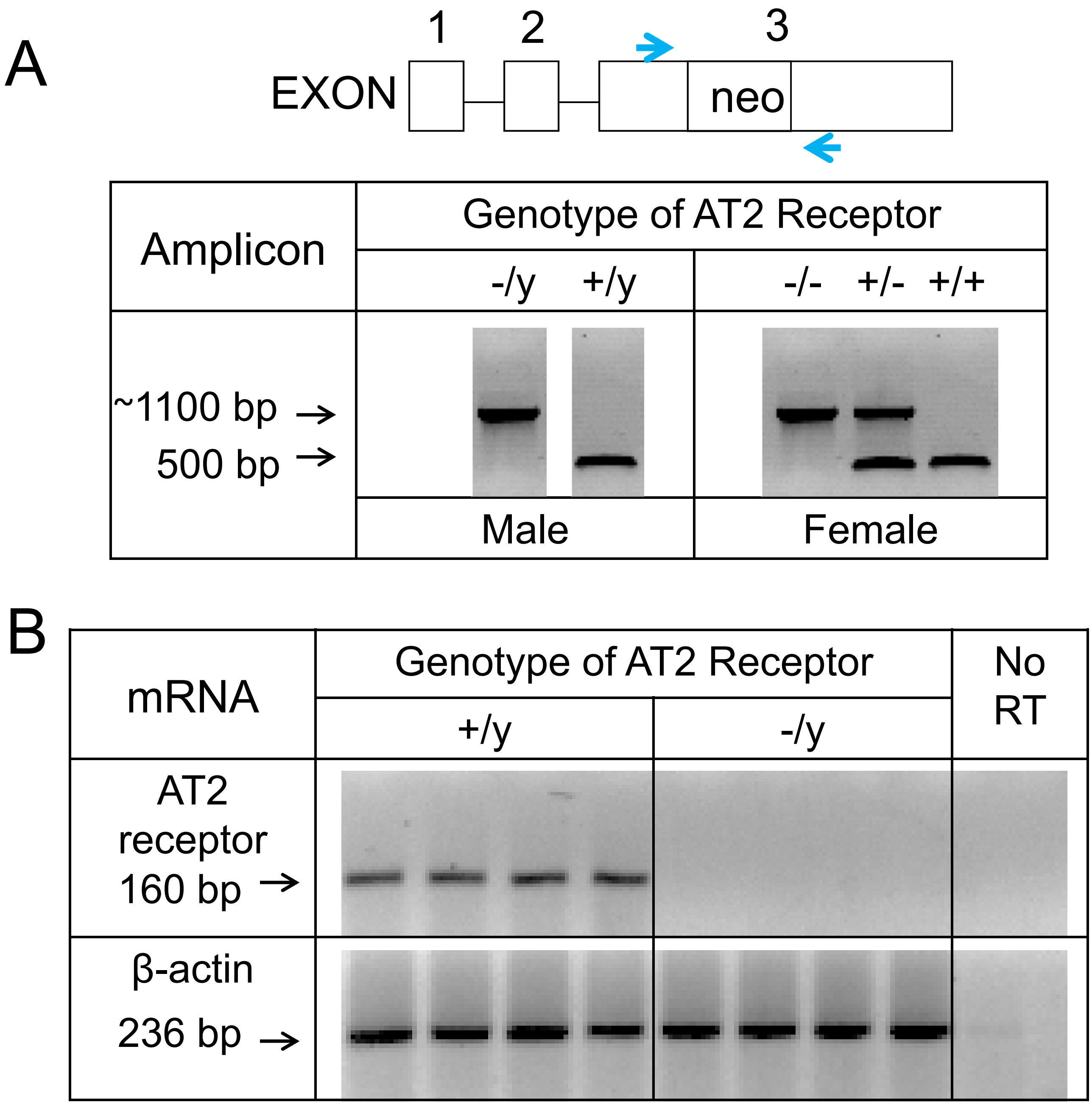

Figure S1. Verification of AT2 receptor deficiency in AT2 -/y mice. (**A**) Genotypes of the AT2 receptor were determined with PCR. Primers (arrows in cyan color) were located upstream and downstream of the neo sequence in exon 3 of the AT2 receptor gene, respectively. Amplicon sizes of wild type and disrupted alleles were 500 bp and ~ 1100 bp, respectively. (**B**) mRNA of AT2 receptor (160 bp) and β-actin (236 bp) was determined by RT-PCR. AT2 receptor mRNA was detectable in extracts of aortas from AT2 +/y mice, but not from AT2 -/y mice. No RT was used as a negative control.
