# Supplementary material for: PD123319 Augments Angiotensin II-Induced Abdominal Aortic Aneurysms through an AT2 Receptor-Independent Mechanism
Source: PLoS One. 2013 Apr 12;8(4):e61849. doi: 10.1371/journal.pone.0061849 (PMC3625148; doi:10.1371/journal.pone.0061849)
Supplement: Figure S2 — Ex vivo images of abdominal aortas. After removal of adventitia, aortas were pinned for photography. Ex vivo diameters were measured using ImagePro Plus software. These selected images represent outer diameters of suprarenal aortas near the mean value of each group. (PDF) [file pone.0061849.s002.pdf]

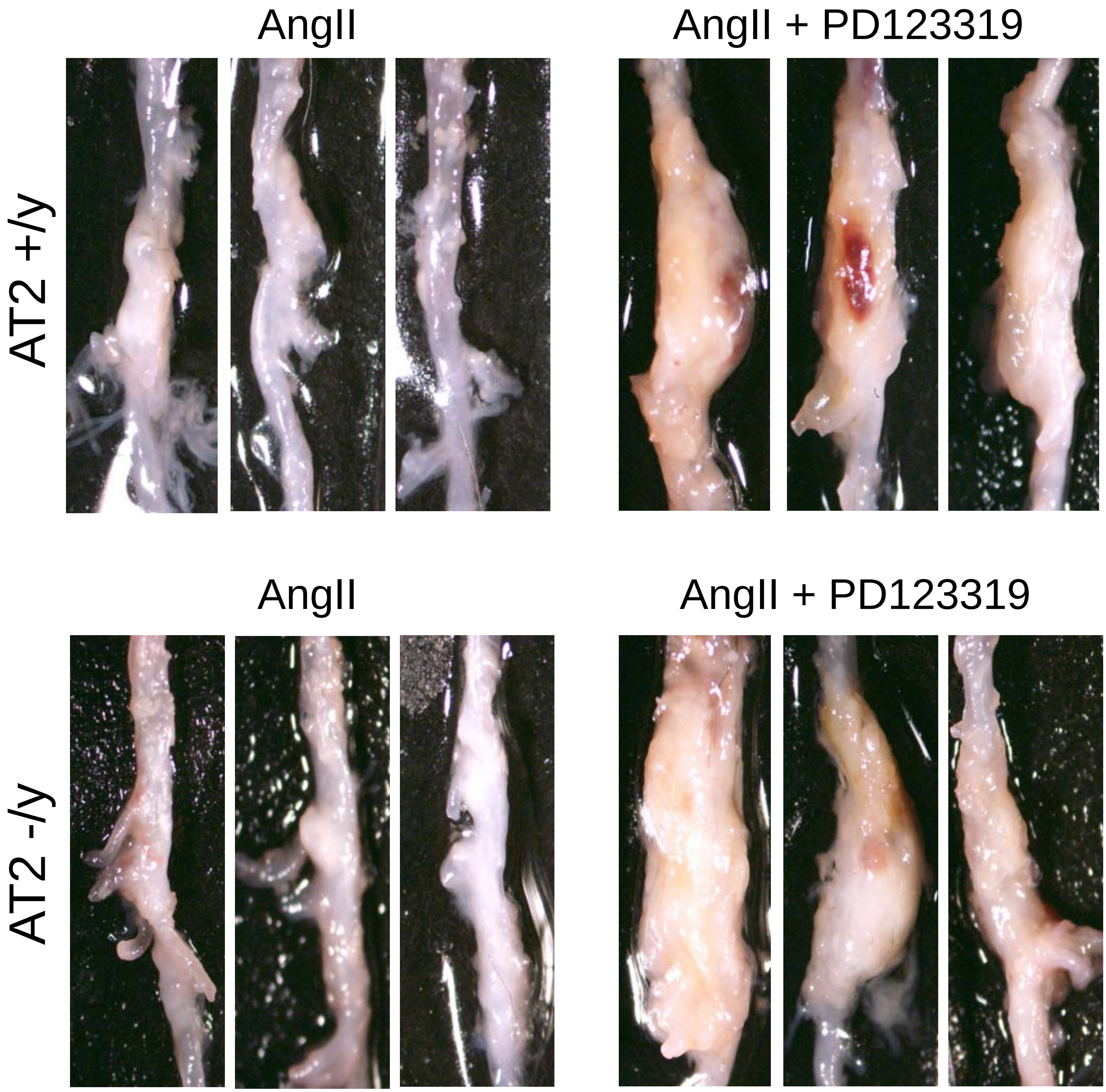

Figure S2. Ex vivo images of abdominal aortas. After removal of adventitia, aortas were pinned for photography. Ex vivo diameters were measured using ImagePro Plus software. These selected images represent outer diameters of suprarenal aortas near the mean value of each group.
