# Supplementary material for: PD123319 Augments Angiotensin II-Induced Abdominal Aortic Aneurysms through an AT2 Receptor-Independent Mechanism
Source: PLoS One. 2013 Apr 12;8(4):e61849. doi: 10.1371/journal.pone.0061849 (PMC3625148; doi:10.1371/journal.pone.0061849)
Supplement: Figure S3 — Pathology of abdominal aortic aneurysms. Suprarenal aortas were sectioned and stained to determine tissue characteristics. Movat's pentachrome staining was performed to visualize structures of aortic tissues. The images of representative sections demonstrate the similar heterogeneity of aneurysmal tissue pathologies in mice infused with PD123319 irrespective of the AT2 genotype. (PDF) [file pone.0061849.s003.pdf]

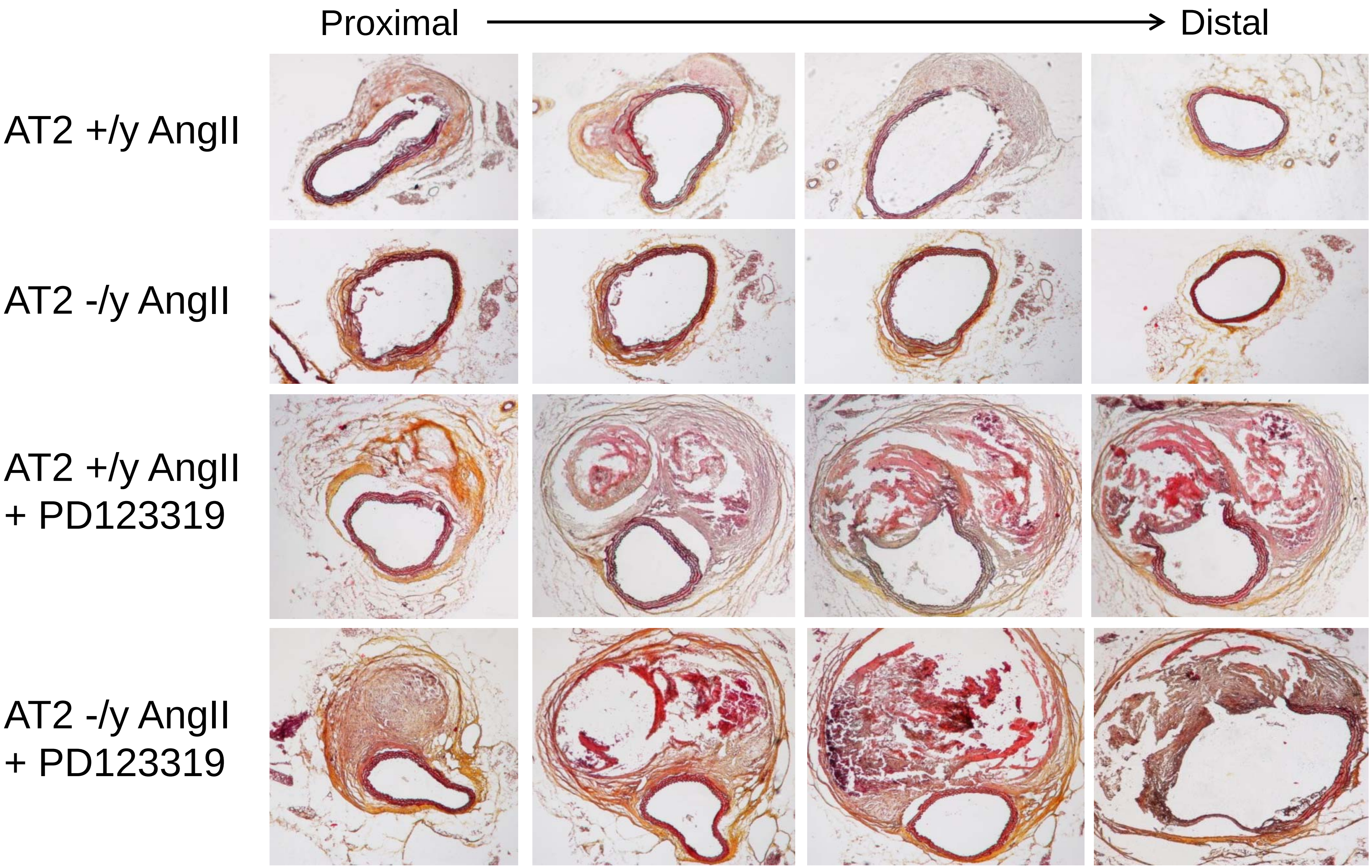

Figure S3. Pathology of abdominal aortic aneurysms. Suprarenal aortas were sectioned and stained to determine tissue characteristics. Movat's pentachrome staining was performed to visualize structures of aortic tissues. The images of representative sections demonstrate the similar heterogeneity of aneurysmal tissue pathologies in mice infused with PD123319 irrespective of the AT2 genotype.
